# IPEX Syndrome: Improved Knowledge of Immune Pathogenesis Empowers Diagnosis

**DOI:** 10.3389/fped.2021.612760

**Published:** 2021-02-22

**Authors:** Federica Barzaghi, Laura Passerini

**Affiliations:** ^1^Department of Paediatric Immunohematology, IRCCS San Raffaele Scientific Institute, Milan, Italy; ^2^Mechanisms of Peripheral Tolerance Unit, San Raffaele Telethon Institute for Gene Therapy (SR-Tiget), IRCCS San Raffaele Scientific Institute, Milan, Italy

**Keywords:** regulatory T cells, IPEX syndrome, FOXP3, immune tolerance, autoimmunity, diagnosis, next generation sequencing

## Abstract

Immune dysregulation, polyendocrinopathy, enteropathy, X-linked (IPEX) syndrome is a rare monogenic autoimmune disease with variable clinical manifestations, ranging from early-onset severe autoimmunity, including enteropathy, eczema, and type 1 diabetes, to late-onset or atypical symptoms. Despite the clinical heterogeneity, the unifying feature of IPEX is mutation of the *FOXP3* gene, which encodes a transcription factor essential for maintenance of thymus-derived regulatory T cells (Tregs). In IPEX patients, Tregs can be present, although unstable and impaired in function, unable to inhibit proliferation and cytokine production of effector T (Teff) cells. Mutated FOXP3 can also disrupt other compartments: FOXP3-deficient Teff cells proliferate more than the wild-type counterpart, display altered T-cell-receptor signaling response, a reduced T-naïve compartment and a skew toward a Th2 profile. Due to *FOXP3* mutations, the frequency of autoreactive B cells is increased and the IgA and IgE production is altered, together with early emergence of tissue-specific autoantibodies. Recently, the awareness of the wide clinical spectrum of IPEX improved the diagnostic tools. In cases presenting with enteropathy, histological evaluation is helpful, although there are no pathognomonic signs of disease. On the other hand, the study of FOXP3 expression and *in vitro* Treg function, as well as the detection of specific circulating autoantibodies, is recommended to narrow the differential diagnosis. Nowadays, Sanger sequencing should be limited to cases presenting with the classical triad of symptoms; otherwise, next-generation sequencing is recommended, given the cost-effectiveness and the advantage of excluding IPEX-like syndromes. The latter approach could be time spearing in children with severe phenotypes and candidate to advanced therapies.

## Introduction

Immune dysregulation, polyendocrinopathy, enteropathy, X-linked (IPEX) syndrome was originally described in the early ‘80s as an early-onset life-threatening systemic autoimmunity in male children (1). The hallmark features comprised enteropathy, severe dermatitis, and autoimmune endocrinopathies (1, 2). Subsequent studies identified the X-linked gene *Forkhead box P*3 (*FOXP3*), a transcription factor member of the forkhead family, as the gene responsible of the disease (3–5). An increasing number of disease-causing variants and a variety of milder clinical forms have been described (2, 6, 7). Increasing clinical awareness was accompanied by growing knowledge of FOXP3 biology on researchers' side. The early evidence that IPEX was associated with T-cell abnormalities (1) and the similarities between the autoimmunity caused by depletion of CD4^+^CD25^+^ regulatory T cells (Tregs) and the phenotype of *foxp3* mutants led to the discovery that FOXP3 dictates Treg function (8, 9). Tregs are a cell subset specialized for immune suppression with a crucial role in maintaining tolerance to self-antigens (10): loss of functional Tregs in IPEX patients is the key pathogenic event leading to uncontrolled autoimmunity (11).

The identification of early signs of multiple autoimmunity in males should prompt clinicians to deeper investigations aimed at identifying other signs of immune dysregulation, possibly sustained by *FOXP3*-mutated Tregs. Therefore, besides histological and serological evaluation, specific study of Tregs may help to validate the suspicion of IPEX syndrome, which, however, needs genetic confirmation.

In this review, we summarize the current knowledge on the function of FOXP3 in the regulation of immunity, with special attention to the immune pathways affected by *FOXP3* mutations. We further highlight the laboratory findings that should drive the differential diagnosis, especially for atypical forms of the disease. We believe that the novel insights into the cellular pathways disrupted by *FOXP3* mutations are of help to speed up diagnosis, optimize the therapeutic approach, and improve clinical outcome.

## Immune Pathogenesis of IPEX Syndrome

FOXP3 is primarily expressed by CD4^+^CD25^+^ Tregs and controls their function and maintenance (10). In IPEX patients, its mutations cause different degrees of Treg dysfunction, ranging from complete lack of suppressive function and skewing to effector phenotype, to partially preserved inhibitory activity (12–16). So far, the impact of few mutations has been characterized in details (13–15); therefore, clear correlation between type and site of the mutation and degree of T-cell functional impairment is still incomplete. Despite its primary function in Tregs, FOXP3 is expressed by other cell types, including effector T (Teff) cells; and its deficiency can affect, either directly or indirectly, their function.

### FOXP3 Mutations and Regulatory T-Cell Fitness

Although first described as the master regulator of Tregs, several studies have demonstrated that FOXP3 is neither necessary nor sufficient to the establishment of the Treg lineage. However, its sustained expression is indispensable for the maintenance of regulatory function, stability, and metabolic fitness (17, 18), that is, full Treg specification.

Indeed, early studies of peripheral T cells in the *scurfy* mice, the natural *foxp3*-mutant, suggested that Foxp3 deficiency resulted in lack of Treg development and complete absence of tTregs (19). Thanks to the use of *foxp3* reporter-gene models (20–22) and to a deeper knowledge of the thymus-derived (t) Treg developmental process (23), it became evident that Treg development in the thymus can occur regardless of FOXP3, although Foxp3-deficient tTregs display functional defects harnessing suppressive ability and peripheral maintenance (20, 24). Through genetic marking of cells actively transcribing a *Foxp3*^*null*^ allele, Gavin and co-workers demonstrated that in the thymus, these marked “Treg-to-be” cells do not undergo negative selection and give rise to a population of Treg precursors, detectable within both mature thymocytes and peripheral CD4^+^ T cells, displaying several common Treg features, including low IL7R expression, *in vitro* anergy, and low cytokine production (20). On the same line, through the generation of a Treg-specific Cre-mediated recombination induced by transcription of a foxp3-null allele, Foxp3-deficient Tregs could be traced *in vivo*: these cells acquired the epigenetic features typical of wild-type (wt) Tregs (22). Similarly, other studies showed that the full development of Treg cells is achieved by the combination of two independent processes, that is, the establishment of specific epigenetic changes and the expression of Foxp3: the two processes are independent since epigenetic features occur before Foxp3 expression and Foxp3 expression can be induced in the absence of Treg methylation patterns (25). Although most of the mechanistic studies were performed in murine models, parallel evidences were found when studying T cells in IPEX patients. First, depending on the type and site of *FOXP3* mutation, normal frequencies of CD4^+^CD25^+^CD127^low^FOXP3^+^ Treg-like cells can be found in the peripheral blood of patients, suggesting that a population of dysfunctional Treg-like cells can emerge from the IPEX thymus and persist in the periphery (7, 26, 27). Second, molecular studies showed that the Treg lineage determining features, including epigenetic imprinting, are acquired during tTreg development prior to FOXP3 expression and occur regardless of FOXP3 function (22). In line with these observations, circulating Treg-like cells of IPEX patients display demethylation of the Conserved Non-coding Sequence 2 (CNS2) in the first intron of the *FOXP3* gene (28). The methylation status of this genomic region, also known as Treg cell-specific demethylated region (TSDR), has been recognized as an epigenetic marker unambiguously identifying Tregs, which allows distinction of tTregs from activated T-conventional cells and *in vitro*-induced Tregs (29, 30). Thus, in IPEX syndrome, *FOXP3* mutations do not hamper the development of tTreg precursors and the emergence of Tregs in the periphery. Third, gene expression profiling of Tregs demonstrated that a FOXP3-independent molecular signature exists (20, 21). Similarly, expression profiling of IPEX Tregs or of FOXP3-mutated transgenic Tregs demonstrated that expression of many Treg signature genes, including TNFRSF18, IKZF2, and LRRC32, are maintained (13, 31), while ectopic FOXP3 expression in T-conventional cells does not fully reproduce natural Treg signature (21, 32) and does not induce Treg-specific methylation pattern (15, 25).

While dispensable for the establishment of the Treg lineage during thymic development, evidences in female *foxp3*^*gfpko*^ transgenic mice supported a fundamental role of Foxp3 in the regulation of Treg homeostasis: Foxp3-dependent downregulation of *Pde3b* expression strictly regulates Treg homeostatic fitness (20). Similar to findings in reporter gene models, functional FOXP3 is indispensable for peripheral maintenance of human Tregs. Indeed, variable frequency of circulating Tregs, ranging from complete absence of FOXP3-expressing cells to increased frequencies as compared with healthy subjects, were found in IPEX patients (7, 26, 33). Furthermore, in healthy female carriers of *FOXP3* mutations, Tregs express exclusively the wt *FOXP3* allele, thus demonstrating that in the periphery, healthy Tregs outcompete those expressing a mutated FOXP3 (34). Similarly, in transplanted IPEX patients with mixed chimerism, donor-derived Tregs display a selective advantage (35, 36). On the same line, in humanized mice (hu-mice) repopulated with FOXP3-KD/KO human hematopoietic stem cells (HSCs), the Treg compartment is uniquely composed of wt FOXP3-expressing cells (37), clearly supporting the idea that in chimeras, FOXP3-deficient Tregs are less fit than their healthy counterpart for long-term survival.

A non-redundant function of FOXP3 is the ability to confer full suppressive function to mature Tregs. The FOXP3-dependent transcriptional program includes genes involved in Treg effector mechanisms, such as Il2ra, Ctla4, Tnfrsf18, and Nrp1 (20, 21). As a consequence, ectopic expression of FOXP3 is sufficient to endow human T-conventional cells with suppressive ability (9, 38, 39), while deregulation of FOXP3 expression leads to loss of suppressive function (40). Of note, stable FOXP3 expression is required not only to confer full Treg activity (39, 41) but also to maintain lineage stability. Although the issue of Treg lineage stability has been actively debated (42), several evidences suggest that Tregs bear an intrinsic plasticity, which allows conversion to Teff cells via losing Foxp3 expression under inflammatory conditions (43, 44). Similarly, in IPEX syndrome, dysfunctional Tregs, unable to properly regulate cytokine production, skew to an effector phenotype, either Th17 or Th2, possibly contributing to the autoimmune damage (16, 45).

FOXP3 has also been described as the “metabolic gatekeeper” of Tregs. While Teff cells are dependent on glycolysis to sustain their metabolism, Tregs preferentially utilize fatty-acid oxidation for proliferation and survival (46). FOXP3 controls Treg metabolism by limiting glycolysis and promoting fatty-acid oxidation via inhibition of the mTORC2 pathway (22). In IPEX syndrome FOXP3 deficiency causes metabolic reprogramming of Tregs with heightened aerobic glycolysis, mainly due to mTORC2 deregulation (22). Indeed, mTORC inhibition improves Treg fitness and partially restores suppressive function of FOXP3-deficient Tregs (22, 31).

**Figure 1:**
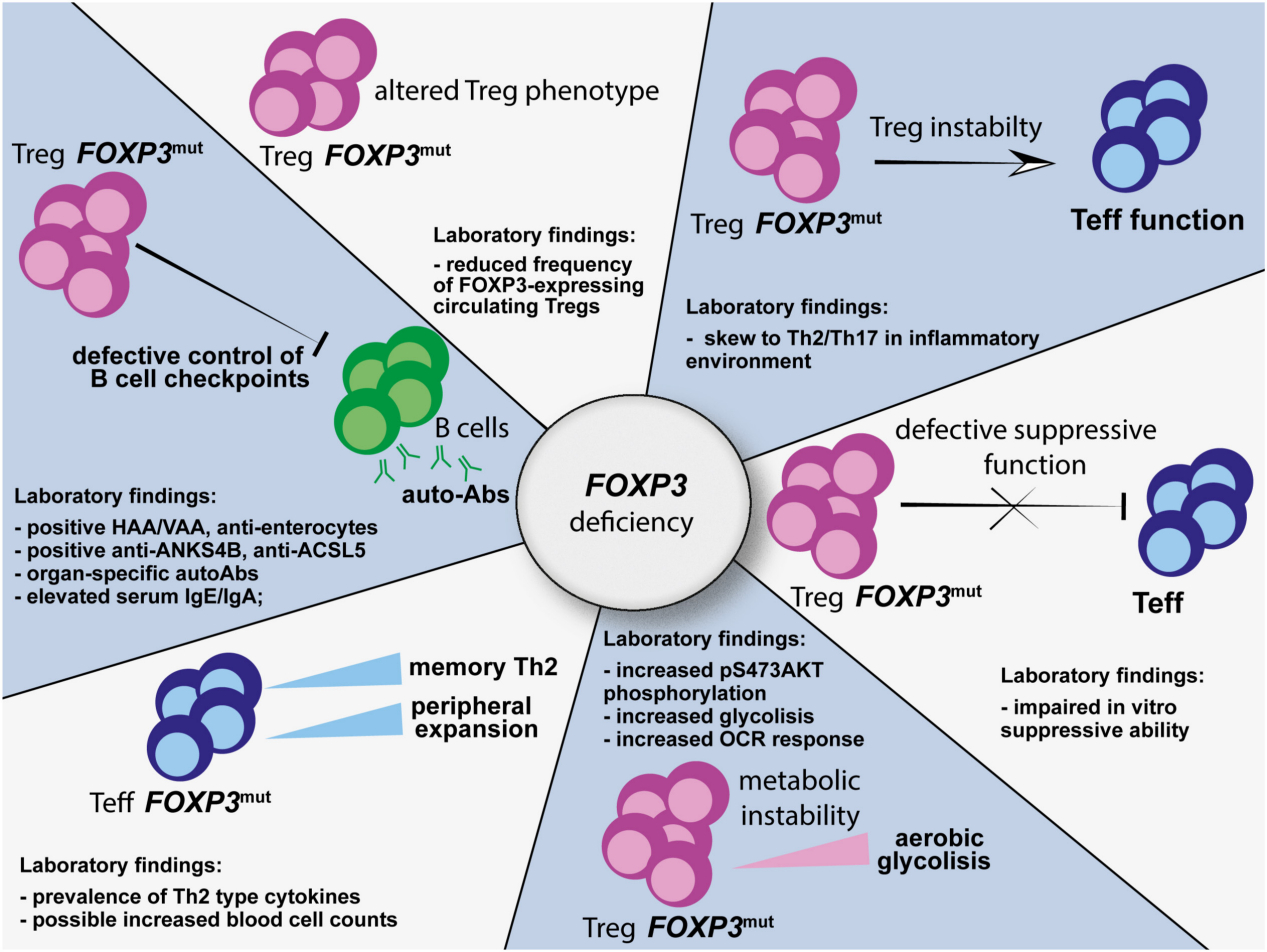
Multiple alterations affecting the Treg, Teff, and B-cell compartments as a consequence of *FOXP3* mutations. HAA, anti-harmonin autoantibodies; VAA, anti-villin autoantibodies; ANKS4B, ankyrin repeat and sterile alpha motif domain containing 4; ACSL5, acyl-CoA synthetase long chain family member 5; HNF4A, hepatic nuclear factor 4 alpha; OCR, oxygen consumption rate; Treg, regulatory T cell; Teff, effector T cell.

Hence, *FOXP3* mutations affect Treg biology at multiple levels (Figure 1). Therefore, in IPEX syndrome, despite commitment to a Treg cell program during thymic development, full Treg specification is not achieved due the lack of full functional properties. Since clear correlation between site of mutation and Treg defects is still unclear, systematic functional and phenotypical analysis of Tregs from patients with immune dysregulation suggestive of IPEX syndrome would help to validate the diagnosis.

### Role of FOXP3 Beyond the Regulatory T-Cell Compartment

Although originally described as exclusively expressed by Tregs (8), transient FOXP3 expression can be induced by activation of T-conventional cells, which do not undergo Treg reprogramming (47). Activated FOXP3-deficient Teff cells displayed increased proliferation and cytokine production and deregulated gene expression, as compared with wt Teff cells, thus indicating a direct effect of FOXP3 expression in Teff cell homeostasis (48). Following studies in FOXP3-KD/KO hu-mice showed that peripheral FOXP3-deficient Teff cells were expanded with a skew toward a memory Th2-type, reminiscent of an aged immune system. The latter hu-mice showed altered thymocyte differentiation, with increased T-cell-receptor (TCR) repertoire diversity and reduced TCR signaling. Data were confirmed by analysis of IPEX patients' peripheral Teff cells (37), thus assigning to FOXP3 deficiency an additional pathogenic mechanism, via shaping of an aberrant Teff compartment.

As indirect consequence of FOXP3 deficiency, B-cell tolerance is also impaired in IPEX: autoreactive B cells accumulate in the periphery, due to altered control of peripheral B-cell tolerance checkpoints by dysfunctional Tregs, resulting in the production of tissue-specific autoantibodies, which can in turn contribute to the clinical manifestations (49–51). Although multiple antigenic specificities have been described (50, 51), the underlying mechanism for loss of checkpoint control by FOXP3-deficient Tregs has not been fully clarified.

These findings support an intrinsic role for human FOXP3 in controlling immune homeostasis in a Treg-independent manner. Therefore, functional analysis and mechanistic studies not only of Tregs, but also of Teff, autoantibodies, and B-cell compartments in patients with suspected IPEX syndrome may help in driving diagnosis (Figure 1) and could potentially indicate targetable pathways to restore immune homeostasis.

## Diagnosis of IPEX Syndrome

IPEX syndrome is a prototype of monogenic autoimmune disease. Due to the autoimmune aggression of different organs, the clinical manifestations at onset may be misleading, since they are common to several inherited disorders (52, 53) mainly attributable but not limited to other inborn errors of immunity (IEI) (54, 55). Hence, the diagnostic process presents two main critical issues. On the one hand, patients with early-onset severe phenotype, mainly related to enteropathy and uncontrolled diabetes, deserve a quick and precise diagnosis, to guarantee an adequate therapy, that is, immunosuppression followed by HSC transplantation (HSCT), whenever feasible. On the other hand, patients displaying atypical, milder, or late-onset autoimmune manifestations can be underdiagnosed for years with consequent slow but constant progression of the damage. In the latter case, a deep characterization of the Treg compartment could drive further investigation, and a wide genetic analysis exploiting next-generation sequencing (NGS) platforms is desirable.

### Clinical Presentation

IPEX syndrome typically involves male patients and encompasses several kinds of clinical presentation, including not only the classical triad [i.e., enteropathy, type 1 diabetes (T1D), and dermatitis], which affects more than a half of IPEX patients, but also autoimmune manifestations involving other target organs (6, 7, 56). While the early onset of enteropathy, autoimmune diabetes, and dermatitis, either alone or in combination, is suggestive of IPEX syndrome, the presence of autoimmunity involving organs other than the typical triad may be misleading, especially if the age of onset is delayed and progression through other autoimmune manifestations either lacks or takes years (6, 53, 56). In the latter cases, evolution over time may help to raise suspicion, since the majority of IPEX patients develop additional autoimmune diseases. Studies on the largest cohorts of IPEX patients (6, 56, 57) report delayed onset above 1 year of age in a limited number of cases ranging from 6% (2/30) (57) to 10.4% (10/96) (6). According to our cohort study (6), the majority of patients with onset between 1 month and 1 year share a clinical picture similar to that of babies below 1 month of age displaying the “classical triad” of symptoms. The same study includes 10 cases (10.4%) of IPEX patients presenting the first symptom over 1 year of age, sometimes consisting of manifestations that are uncommon at onset, such as nephritis or hepatitis. Single case reports have described patients presenting typical symptoms, such as intractable diarrhea, but with onset delayed to early childhood (1, 58, 59) or even later to adolescence (1, 60). These patients experienced mild enteropathy (loose stools or recurrent/remittent diarrhea) as first manifestation, thus resulting in a significant diagnostic delay. Other studies reported onset above 1 year of life with mild phenotype, such as alopecia and dermatitis (61), and arthritis involving one or more joints (62, 63).

Additional atypical and rare manifestations of IPEX syndrome include autoimmune pancreatic exocrine insufficiency (33, 64–66), gastritis (33, 65, 67), kidney disease (68–72), interstitial lung disease (62, 73, 74), or steroid-responsive pneumonia (63).

Intractable diarrhea can lack, however, the association of manifestations likely due to immune dysregulation (e.g., dermatitis, alopecia, cytopenia, and nephropathy), and the presence of failure to thrive should prompt immunological investigations (75). Similarly, any autoimmune endocrinopathy (besides T1D), arising in males at birth or very early in life, should prompt an immunological follow-up to monitor the development of any other autoimmune manifestation (76). Also patients displaying severe allergic conditions in association with other autoimmune symptoms should raise the suspicion of IPEX syndrome (70, 77–79). The increasing number of diagnosed patients with atypical forms suggests that the expressivity of the disease is variable and likely influenced by epigenetic factors or modifying genes.

The diagnosis of IPEX syndrome relies on the collaboration of several specialists. The first step to improve patients' referral is to improve the awareness of each physician that could come to face the clinical signs. Clinical reports taught us that a timely diagnosis is crucial, since supportive and immunosuppressive therapies, although life-saving in the acute phase, are not *per se* curative (6). An early genetic diagnosis may allow a prompt donor search for HSCT, which would halt disease progression, restoring a functional Treg compartment.

### Laboratory Findings

Laboratory investigations in IPEX patients should aim at excluding other IEI that can present with enteropathy or other autoimmune manifestations and at driving differential diagnosis with IPEX-like syndromes and other Treg-opathies (52, 80, 81).

First-line investigations, for example, complete blood cell counts, lymphocyte subsets, lymphocyte proliferative response to mitogens, and immunoglobulin (Ig) plasmatic levels, are usually associated with screening for dysfunction of potential target organs, for example, nutritional indexes, glycated hemoglobin, fasting glucose, and kidney and hepatic function. In IPEX patients, the mentioned laboratory investigations can be normal at onset. Leukocytosis, if present, is due to an increase in lymphocytes, although subset composition and the proportion of naive and memory T cells are not deeply affected, despite immune dysregulation (2, 7, 53, 56). Of note, the CD4/CD8 ratio was reported as increased (53). Proliferative response to mitogens is usually present (53). Serum IgG and IgM are generally normal or low if protein-losing enteropathy is present (33). The identification of eosinophilia, cytopenias, or increased IgE and IgA are common features of IPEX patients (2, 6). In the presence of abnormalities in Ig levels, lymphocyte counts, or function, the differential diagnosis will include both immunodeficiencies of the T and/or B cell subsets or other IPEX-like diseases (6, 7, 56). TCR repertoire is polyclonal, and the *in vitro* cytokine production shows a skew to a Th2-type profile (3, 26, 61).

In IPEX patients with either enteropathy or gastritis, histological evaluation is essential to exclude other etiologies, but it is not diagnostic: the inflammatory infiltrates are usually polymorphic with predominance of lymphocytes and eosinophils. Villous atrophy is typical, although not pathognomonic. Other unspecific inflammatory lesions (ulcers, crypt hyperplasia, and abscesses) can be present. Metaplastic and dysplastic lesions of the gastric mucosa are rare complications (6, 31, 70, 71).

**Figure 2:**
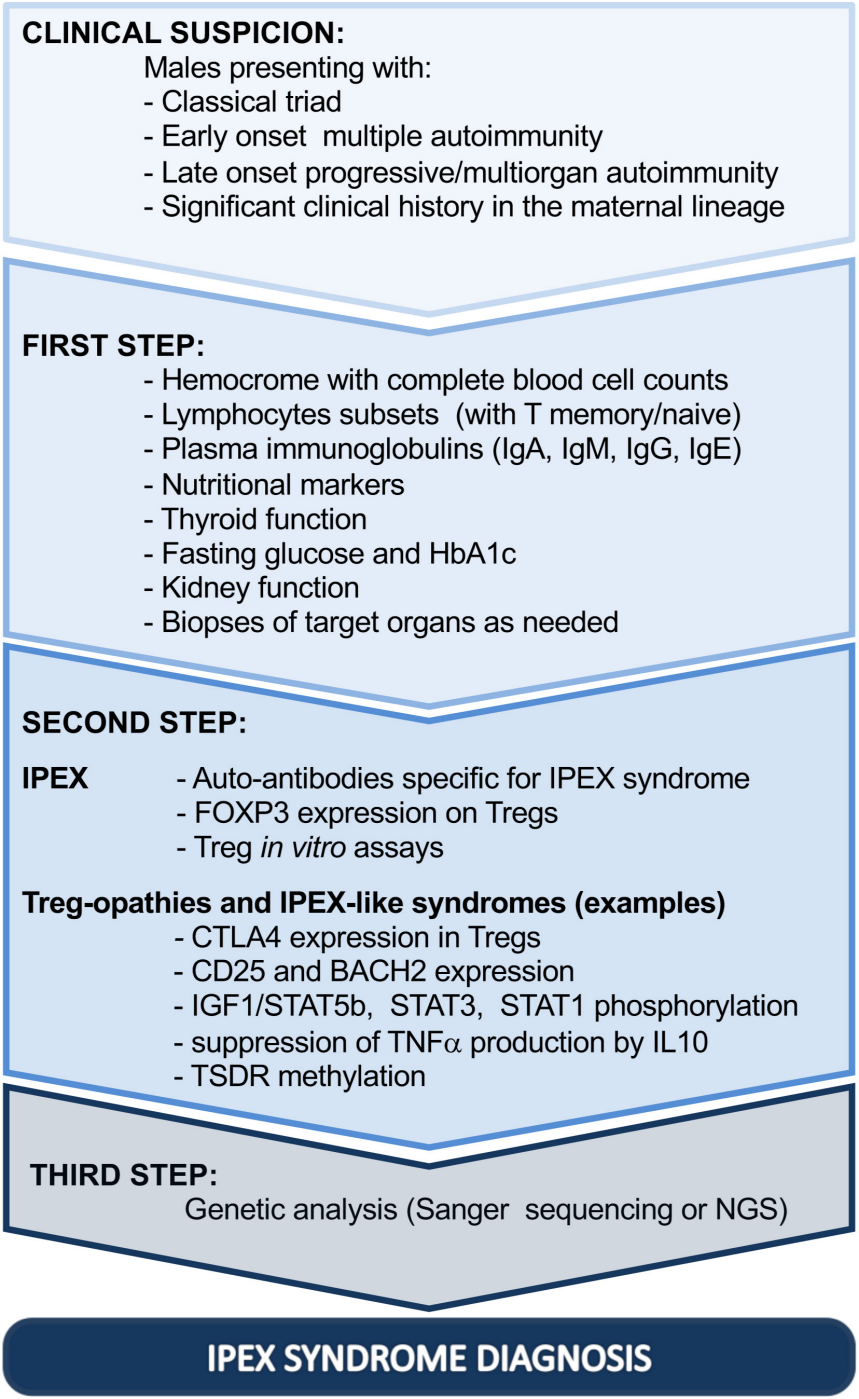
Diagnostic steps driving toward IPEX syndrome. Ig, immunoglobulin; HbA1c, glycated hemoglobin; FOXP3, forkhead box protein P3; CTLA4, cytotoxic T-lymphocyte protein 4; BACH2, BTB Domain And CNC Homolog 2; TNF, tumor necrosis factor alpha; IL-10, interleukin 10; STAT1, signal transducer and activator of transcription 1; STAT3, signal transducer and activator of transcription 3; IGF1, insulin-like growth factor I; STAT5b, signal transducer and activator of transcription 5b; TSDR, Treg cell-specific demethylated region; NGS, next-generation sequencing; IPEX, immune dysregulation, polyendocrinopathy, enteropathy, X-linked.

Similarly, skin histopathological changes are variable and non-specific, ranging from atopic/psoriasiform dermatitis to uncommon allergic, autoimmune, or infectious complications (61, 67, 82, 83). Thus, histological evaluation of the gut and skin can highlight the autoimmune etiology without necessarily confirming the diagnosis (Figure 2, step 1).

Second-line investigations should include, if available, specific tests aimed at identifying the typical immunological alterations of IPEX syndrome. Since Treg dysfunction alters the humoral response, specific autoantibodies detectable in patients' serum are possible biomarkers of the disease in the presence of gastrointestinal manifestations. IPEX patients typically harbor circulating autoantibodies against harmonin (HAA), a 75-kDa protein also known as USH1C, expressed in the intestinal epithelia and renal tubules (70, 71, 77, 84). Although the assay is not widely accessible, positivity of serum or plasma samples to HAA is highly suggestive of the diagnosis (85). Moreover, circulating autoantibodies against villin (VAA, actin-binding 95-kDa protein), an antigen with the same histological distribution of harmonin, have been described as concomitant or alternative to HAA (85, 86). All IPEX patients, positive for either HAA or VAA, tested positive for anti-enterocyte antibodies by indirect immunofluorescence (85). Moreover, new autoantibody targets have recently been identified in three structural proteins: Ankyrin Repeat And Sterile Alpha Motif Domain Containing 4 (ANKS4B), which interacts with harmonin in the intestinal microvilli; Acyl-CoA Synthetase Long Chain Family Member 5 (ACSL5), a regulator of enterocytes proliferation; and Hepatic Nuclear Factor 4 Alpha (HNF4A), a regulator of the intestinal and renal epithelium differentiation (50). Nevertheless, presence of circulating autoantibodies to the latter targets has a weaker association with IPEX syndrome as compared with HAA, while they could support the diagnosis in rare cases of HAA/VAA negativity (50). Recently, elevated levels of plasmatic neutralizing autoantibodies against interferon-α have been described in a cohort of IPEX patients (87).

A variety of other autoantibodies have been detected in most patients, and their presence in circulation usually correlates with signs of pathology in specific target organs (e.g., anti-insulin, anti-pancreatic islet cells, anti-glutamate decarboxylase, anti-thyroglobulin, anti-microsome peroxidase, Coombs test, anti-platelets, anti-neutrophils, anti-smooth-muscle, and anti-liver-kidney-muscle antibodies), although their production may be a sign of immune dysregulation without a related pathological evidence (6, 88). Serological as well as histological markers are indicative of the diagnosis only when considered in combination with the clinical manifestations.

Moreover, second-line investigations should consider the possible effect of *FOXP3* mutations in Tregs, which result largely in dysregulation in IPEX patients, as previously illustrated. The proportion of Tregs, evaluated by flow cytometry, may be highly variable: low or absent FOXP3-expressing cells can be considered as highly suspicious for IPEX syndrome; however, several *FOXP3* mutations demonstrate deleterious effects on the protein function, without preventing protein expression (27, 33). Especially in these cases, *in vitro* evaluation of Treg function may dispel any doubt on Treg dysfunction, even though Treg suppression assays are not standardized (Figure 2, step 2). It is important to underline that defects in FOXP3 expression or in Treg function can be shared by different Treg-opathies, as defined by IUIS 2019, due to mutations in genes with an impact on Treg fitness and/or function (*CD25, CTLA4, LRBA, STAT3, BACH2, CD122, DEF6*, and *FERMT1*) (80, 81). Clinically, they resemble IPEX syndrome, especially if manifesting with enteropathy and cytopenias. However, Treg-opathies other than IPEX syndrome show higher prevalence of infections and/or lymphoproliferation, which are indeed rather uncommon in IPEX syndrome (80). Moreover, some diseases of immune dysregulation fall in differential diagnosis with IPEX syndrome mainly due to the fact that patients can present severe inflammatory bowel disease and other IPEX-like autoimmune features, but they do not show tTreg cell impairment (mutations in *ITCH, TPP2, JAK1, PEPD, IL10*, and *IL10R*) (52, 80). Finally, some IEI, also classified as combined immunodeficiencies, can share with IPEX syndrome signs of enteropathy or widespread autoimmunity early in life, with or without Treg impairment, but with clear defects in other T- and B-cell subsets and additional manifestations strictly related to the consequent immunodeficiency (e.g., mutations in *RAG1, RAG2, CD3G, NEMO, WASP, ARPC1B, XIAP, NLRC4, STAT5b, NFKBIA*, and *TTC7A*) (52, 80).

Analysis of the demethylation status of the TSDR, possibly combined with T cell-Specific-Demethylated-Region (TLSDR) analysis, in order to exclude a bias related to lymphopenia can be considered as additional tools to discriminate IPEX-like patients, who display a significantly reduced percentage of peripheral Tregs, as compared with healthy subjects, patients with IPEX syndrome, and patients with different autoimmune and autoinflammatory diseases (28). Although few patients were investigated, based on TSDR analysis on the peripheral blood of IPEX patients, we reported that Tregs, although FOXP3-mutated, are present in a higher amount than those detected in IPEX-like patients and healthy subjects (28). This method has been recently applied also to dried blood spots of a newborn and an infant carrying *FOXP3* mutations: similarly to previous report (28), the percentage of Tregs within CD3^+^ T cells increased compared with that in non-affected healthy newborns (89). Although the finding needs further validation, quantification of TSDR and TLSDR is a promising approach for larger application in patients with immune dysregulation during the diagnostic workup, integrating flow cytometric evaluation, especially in the presence of inflammation, and standardizing the quantification of Tregs.

As further support in the diagnostic process, the use of flow cytometry to investigate biological alterations related to other IEI can fill the gap between conventional immunological tests and genetic analysis (90). Indeed, based on the clinical features of the patient, a selection of functional and phenotypical tests can be performed to narrow the differential diagnosis and to anticipate and guide the genetic analysis especially when NGS is ongoing. Some examples are provided in Figure 2, step 2.

### Definitive Molecular Diagnosis

IPEX syndrome has heterogeneous clinical presentation and evolution; hence, finding *FOXP3* mutation is at the same time challenging and paramount for the child prognosis. Both in case of severe rapidly evolving symptoms and in case of chronic slowly progressive clinical course, the risk–benefit evaluation of the therapeutic options could be difficult without a genetic diagnosis.

Given the mentioned phenotypical overlap of IPEX syndrome with a series of IEI (with or without immune dysregulation), Sanger sequencing of the *FOXP3* gene is usually applied to selected cases, such as classical triad of symptoms, neonatal T1D, and intractable diarrhea in neonates with significant family history in the maternal lineage (including affected males, fetal hydrops, and multiple miscarriages of male fetuses) (1, 4, 91).

In neonatal forms presenting with isolated symptoms, such as intractable diarrhea or widespread dermatitis, without significant family history, structural diseases of the gut or skin epithelium as well as other IEI could be included in the differential diagnosis, before the full-blown clinical picture is evident. Hence, the contemporary analysis of multiple genes by NGS would be the approach of choice, given the cost-effectiveness and the advantage of excluding simultaneously multiple diseases. Moreover, this approach could be time-spearing in children with severe phenotypes and candidate to HSCT or advanced therapies, currently under development (38, 92, 93). The choice between targeted gene sequencing and whole-exome sequencing depends on the single center practice. However, in order to guide the analysis and prioritize candidate genes, IEI typically associated with immune dysregulation should be investigated (52, 80). Among them, Treg-opathies related to gain-of-function mutations of *STAT1* or *STAT3* and deficiency of LRBA have been recently reported as most frequently associated with IPEX-like diseases (7, 56). A similar phenotype can be determined by mutations in *IL2RA, CTLA4, BACH2, STAT5B*, and *IL10*/*IL10R* (7, 56, 81).

Although genetic analysis is useful to support therapeutic choices, genotype–phenotype correlation remains debated (2, 6, 7). Therefore, it cannot be used currently to foresee disease progression (Figure 2, step 3).

## Conclusive Remarks

Lessons from nearly 20 years of research on basic biology and patients taught us that mutations in the *FOXP3* reduce suppressive function of Tregs due to deregulation of key effector genes, but also show consequences on other lymphocyte subsets. In this light, the study of Tregs and specific autoantibodies in patients with suggestive clinical phenotype can contribute to steer genetic diagnosis and to choose the most suitable method for genetic analysis, since timing is crucial and a prompt intervention would revert the autoimmune process, thus preserving the target organ from fatal injury.

## Author Contributions

Both authors wrote the manuscript. Both authors contributed to the article and approved the submitted version.

## Conflict of Interest

The authors declare that the research was conducted in the absence of any commercial or financial relationships that could be construed as a potential conflict of interest.
